# Clinical Factors Associated with Atrial Fibrillation Detection on Single-Time Point Screening Using a Hand-Held Single-Lead ECG Device

**DOI:** 10.3390/jcm10040729

**Published:** 2021-02-12

**Authors:** Giuseppe Boriani, Pietro Palmisano, Vincenzo Livio Malavasi, Elisa Fantecchi, Marco Vitolo, Niccolo’ Bonini, Jacopo F. Imberti, Anna Chiara Valenti, Renate B. Schnabel, Ben Freedman

**Affiliations:** 1 Cardiology Division, Department of Biomedical, Metabolic and Neural Sciences, University of Modena and Reggio Emilia, Policlinico di Modena, 41125 Modena, Italy; nanni.malavasi@gmail.com (V.L.M.); elisa.fantecchi@gmail.com (E.F.); marco.vitolo@unimore.it (M.V.); bonini.niccolo93@gmail.com (N.B.); jacopo.imberti@hotmail.it (J.F.I.); annachiara.valenti@gmail.com (A.C.V.); 2 Cardiology Unit, “Card. G. Panico” Hospital, 73039 Tricase, Italy; dr.palmisano@libero.it; 3 Clinical and Experimental Medicine PhD Program, University of Modena and Reggio Emilia, 41125 Modena, Italy; 4 German Cardiovascular Research Center (DZHK), Partner Site Hamburg/Kiel/Lübeck, University Heart and Vascular Centre, 20251 Hamburg, Germany; r.schnabel@uke.de; 5 Heart Research Institute, Charles Perkins Centre, and Concord Hospital Cardiology, University of Sydney, Sydney 2006, Australia; ben.freedman@sydney.edu.au

**Keywords:** age, atrial fibrillation, risk stratification, stroke

## Abstract

Our aim was to assess the prevalence of unknown atrial fibrillation (AF) among adults during single-time point rhythm screening performed during meetings or social recreational activities organized by patient groups or volunteers. A total of 2814 subjects (median age 68 years) underwent AF screening by a handheld single-lead ECG device (MyDiagnostick). Overall, 56 subjects (2.0%) were diagnosed with AF, as a result of 12-lead ECG following a positive/suspected recording. Screening identified AF in 2.9% of the subjects ≥ 65 years. None of the 265 subjects aged below 50 years was found positive at AF screening. Risk stratification for unknown AF based on a CHA_2_DS_2_VASc > 0 in males and >1 in females (or CHA_2_DS_2_VA > 0) had a high sensitivity (98.2%) and a high negative predictive value (99.8%) for AF detection. A slightly lower sensitivity (96.4%) was achieved by using age ≥ 65 years as a risk stratifier. Conversely, raising the threshold at ≥75 years showed a low sensitivity. Within the subset of subjects aged ≥ 65 a CHA_2_DS_2_VASc > 1 in males and >2 in females, or a CHA_2_DS_2_VA > 1 had a high sensitivity (94.4%) and negative predictive value (99.3%), while age ≥ 75 was associated with a marked drop in sensitivity for AF detection.

## 1. Introduction

Atrial fibrillation (AF) is a very common arrhythmia, with a prevalence and incidence that increase with advancing age [1,2,3,4]. AF is associated with a 5-fold increase in the risk of thromboembolism and stroke and is detected in about 20% of strokes, both in form of symptomatic or asymptomatic arrhythmia [3,5,6,7,8,9,10,11]. Underdiagnosed and so undertreated AF, as in case of asymptomatic or unknown arrhythmia, exposes patients to the risk of thromboembolic events and their downstream complications [12]. Thus, AF screening, by pulse palpation or using devices targeted to AF detection through specific sensors or electrocardiographic recording, is advisable [7,13,14]. Several initiatives for AF screening have been carried out in Europe, North America, and other areas, with substantial differences related to the type of screening (opportunistic vs. systematic), the setting of the intervention (primary care, pharmacies, community, etc.), methods used for AF detection (photoplethysmography, single-lead ECG recording or other sensors), characteristics of the screened population (age and risk factors of participants) [7,13,14,15,16]. The aim of the present study was to assess the prevalence of unknown AF among adults with no previous history of AF, during a single-time point AF screening, performed on the occasion of meetings or social recreational activities organized by groups of volunteers and associations for promoting healthy behaviors and wellness. Additionally, we aimed to assess which clinical variables, among those usually included in clinical risk scores for AF management, could provide a higher chance of diagnosing a new AF, alone or in combination.

## 2. Materials and Methods

Data were collected anonymously during 20 initiatives held by volunteers, patient groups and associations for promoting healthy behaviors and wellness. Two to four physicians voluntarily participated in every initiative and explained to potential participants the purpose and the implications of AF screening.

The number of participants who agreed to undergo the AF screening at each session varied between 80 and 600. Participants were at least 18 years and provided informed consent, after detailed information on the reasons for searching AF and on the implications of its detection. The study protocol was approved by the local Ethics Committee (N. 692/2020 Comitato Etico AVEN) for retrospective analysis of collected data. Subjects who reported a known history of AF or those with an implanted cardiac implanted electronic device (pacemaker or defibrillator) were not included. Participation of subjects aged 65 years or older was encouraged, but younger subjects were accepted too. Before testing, the voluntary medical personnel performed a brief interview with each participant in order to collect anonymized data regarding patients’ characteristics, as well as factors included in the CHA_2_DS_2_-VASc score [14,17,18].

When evaluating thromboembolic risk, most international guidelines recommend the use of the CHA_2_DS_2_-VASc score [18]. However, the Australian guidelines recommend a modified CHA_2_DS_2_-VA score (the sexless CHA_2_DS_2_-VASc score) while the Canadian guidelines adopt the Congestive Heart Failure, Hypertension, Age 65 years, Diabetes, Stroke/Transient Ischemic Attack (CHADS-65 also known as CCS algorithm). For this reason, we included the most common risk stratification scores recommended by different Guidelines in order to make results applicable to European and non-European realities [18].

Since the aim of our study was to investigate the possibility to implement and test the efficacy of an opportunistic screening of AF during meetings or social recreational activities organized by patient groups or volunteers regardless of symptoms assessment, we did not include symptoms among the criteria for patient recruitment, in line with usual policy for screening of AF initiatives, targeted to include all the subjects in whom AF had not been diagnosed previously [7,16,19].

For testing, we used the MyDiagnostick bar device (Applied Biomedical Systems BV, Maastricht, The Netherlands), a single-lead ECG device commonly used in AF screening initiatives, which returns a green or red light according to the absence or presence of rhythm irregularities suspected for AF, through an automatic analysis of tracings associated proven to be reliable in terms of sensitivity and specificity [20,21]. The MyDiagnostick device has a shape of a stick (length 26 cm, diameter 2 cm) with electrodes at both ends and it automatically switches on when held by the patient. Rhythm analysis requires that the individual simply holds the device in both hands for 60 s. The device turns red, in case of rhythm irregularities suspected for AF, or green, indicating a normal cardiac rhythm. The MyDiagnostick can store up to 140 ECG Lead I strips lasting 1 min each. The device can be connected vis USB to a computer and interrogated immediately to show the last recorded ECG strip.

Enrolled patients were invited by voluntary medical personnel to hold the MyDiagnostick device for 1 min. In case of a red alarm, indicating an irregular tracing suspected to be AF, a 12-lead ECG was performed within 24 h and interpreted by a cardiologist to confirm the presence of AF. AF was diagnosed only when confirmed at the 12-lead ECG tracing [22]. The same physicians involved in AF screening were responsible for organizing the access to the 12-lead ECG within 24 h, in every case of red alarm at the MyDiagnostick device. All the subjects with AF diagnosed at the 12-lead ECG were directly referred to a cardiologist, for a complete clinical evaluation, according to usual practice, and for prescription of anticoagulants, when appropriate. Data were analyzed considering the diagnosis of this arrhythmia on the 12-lead ECG performed after detection of an irregular rhythm by MyDiagnostick device.

The primary analysis was performed in the whole group of participants, focusing on the detection of AF as confirmed by 12-lead ECG, and on the identification of factors associated with a higher likelihood of AF detection. The secondary analysis was targeted to the subgroup of subjects aged ≥65 years.

### Statistycal Analysis

Continuous variables were expressed as median and interquartile range [IQR]. Categorical variables were reported as number of patients and percentages.**** Data were analyzed with univariate logistic regression and individual variables included in the CHA_2_D_2_VASC score with a *p*-value < 0.10 were inserted in a multivariate logistic regression model with the correct identification of AF as dependent variable. Sensitivity, specificity, positive predictive value (PPV) and negative predictive value (NPV) were also assessed with an incremental dichotomization of the scores to detect the best values for each risk score. The receiver operating characteristic (ROC) curve was used to estimate the optimal cut-off value for age in detecting AF in our population. The best fitting value was determined according to the Youden Index.

A *p*-value < 0.05 was considered significant. IBM SPSS v.21 (IBM Corp., Armonk, NY, USA) was used for the analysis.

## 3. Results

A total of 2814 subjects with a median age of 68 years (interquartile range (IQR) 61–74) underwent screening for AF. The general characteristics of the subjects and the results of the screening are shown in Table 1. Overall, 56 subjects (2.0%) were found to have AF, as a result of a 12-lead ECG following a positive recording with the MyDiagnostick device. In other 53 subjects with possible AF on the MyDiagnostick, AF was not confirmed at 12-lead ECG and patients were considered as AF-free.

No one of the 265 tested subjects aged below 50 years was diagnosed with AF. Subjects with a positive screening differed from those with a negative screening test for several variables, as shown in Table 1, with the results of univariate comparisons.

**Figure 1:**
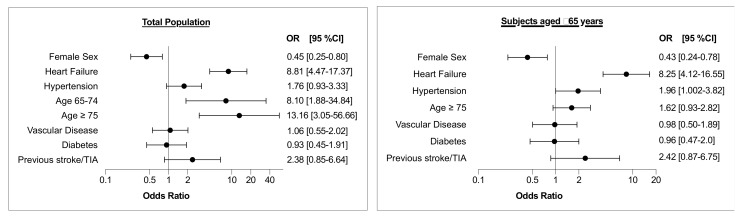
Results of multivariate analysis evaluating the characteristics of study participants with regard to screen-detected AF, as confirmed by 12-lead ECG. Vascular disease includes CAD, previous myocardial infarction and/or peripheral artery disease. Left panel: Total population; Right panel: Subset of subjects aged ≥ 65 years. TIA = transient ischemic attack; OR = odds ratio; CI = confidence interval.

The results of multivariate analysis evaluating the characteristics of study participants concerning screen-detected AF, as confirmed by 12-lead ECG, are shown in Figure 1 (left panel). Heart failure, age ≥ 75 years, as well as age 65–74 years were independently associated with screen-detected AF, while female sex was inversely related to AF detection.

The analysis of results on the whole population, by applying different selection criteria, based on variable cut-off values of CHA_2_DS_2_VAsc score, CHA_2_DS_2_VA score, CHADS_2_ score and CHADS_2_65, or according to two age thresholds, taking into account identification of subjects with screen-detected AF, as confirmed by 12-lead ECG, is shown in Table 2.

The results in the whole tested population showed that stratification based on a single sex-independent factor (e.g., CHA_2_DS_2_VASc > 0 in males and >1 in females or CHA_2_DS_2_VA > 0, or CHADS_2_ > 0) identified subjects with screen-detected AF with a sensitivity higher than 90%, and a negative predictive value of at least 99.6%.

Similar sensitivity and negative predictive value were achieved by using an age threshold of 65 years as a risk stratifier, but a lower sensitivity was conversely associated with a threshold of 75 years. As shown in Figure S1, the ROC curve showed that age alone had a modest predictive ability for detecting AF (area under the curve [AUC]: 0.721, 95% CI: 0.665 to 0.778). According to Youden Index, age > 69 had the highest sensitivity (78.6%) and specificity (55.8%) values. However, using a cut-off of ≥65 years old, we found a higher sensitivity (96.4%).

The characteristics of subjects aged ≥ 65 years are shown in Supplementary Table S1. Among subjects aged ≥ 65 years AF, as confirmed by 12-lead ECG, was found in 2.9% of the subjects who underwent AF screening. The results of multivariate analysis evaluating the characteristics of study participants aged ≥ 65 years with regard to screen-detected AF, as confirmed by 12-lead ECG, are shown in Figure 1 (right panel). Heart failure and hypertension were independently associated with screen-detected AF, while female sex was inversely related to arrhythmia detection.

Table 3 shows the analysis of the screening process, by applying different selection criteria, based on variable cut-off values of CHA_2_DS_2_VAsc score, CHA_2_DS_2_VA score, CHADS_2_ score and CHADS_2_65, or according to age ≥ 75, among the 1848 subjects aged ≥ 65 years. A CHA_2_DS_2_VASc > 1 in males and >2 in females, or a CHA_2_DS_2_VA > 1, resulted in the highest sensitivity and negative predictive value while age ≥ 75 was associated with a marked drop in sensitivity.

## 4. Discussion

The present study highlights that opportunistic screening for AF performed during initiatives promoted by volunteers, patient groups and associations for sensitization on healthy behaviors and wellness, using a single-time point method with a simple hand-held ECG device, results in the detection of AF in 2.0% of the whole screened population and 2.9% of subjects aged ≥ 65 years. Moreover, our results highlight that the use of simple parameters, such as age or currently used risk scores, specifically the CHA_2_DS_2_VASc score (>0 in males and >1 in females) or CHA_2_DS_2_VA > 0, may improve patient targeting and AF detection during screening initiatives. Although younger age was not an exclusion criterion, our study suggests that age ≥ 65 may allow to achieve a considerable sensitivity (96.4%) but in the whole population a slightly better result (up to 98.2% in sensitivity) can be obtained considering a CHA_2_DS_2_VASc > 0 in males and >1 in females (or CHA_2_DS_2_VA > 0), as an alternative to simply adopt the age ≥ 65 cut-off. The practical implications are that the same criteria that indicate the need for long-term anticoagulation in patients with documented AF, can be proposed for selecting the candidates for AF screening, in parallel with the traditional age cut-off of 65 [14]. Targeted screening of subjects at higher risk of having undetected and unknown AF has the potential to significantly reduce the number needed to screen [23,24]. Many consensus documents and guidelines recommend opportunistic screening for subjects aged ≥ 65 [7,13,14,25], but also suggest systematic ECG screening in subjects aged ≥ 75 or at high risk of stroke [14]. Limiting the analysis to subjects aged ≥65 years, CHA_2_DS_2_VASc > 1 in males and >2 in females, or CHA_2_DS_2_VA > 1, are associated with the highest sensitivity and negative predictive value for AF detection. Conversely, according to our data, adopting an age cut-off of ≥75 may be associated with a low sensitivity (around 50%) in a single-time point ECG screening because many cases, in particular in the decade 65 to 75 years are missed. In our study, age alone had a modest predictive ability for detecting AF, with age > 69 having the highest sensitivity (78.6%) and specificity (55.8%) values. However, in this type of screening initiatives, the aim is to reach the highest sensitivity value even accepting a loss in specificity. Using a cut-off of ≥65 years old we found that sensitivity was up to 96.4%, confirming, as suggested by the 2020 ESC AF guidelines [14], that opportunistic screening for AF is recommended in patients ≥ 65 years of age. In the meta-analysis by Lowres et al. [13] AF detection rates with screening were 0.73% between 65 and 69 and 1.09% between 70 and 74, respectively, taking into account a wide range of screening methods. The age threshold of 75 years was suggested in the STROKESTOP study in which more intensive ECG recordings were performed (patient-activated ECG for 30 s at least twice daily for 2 weeks with a handheld ambulatory ECG recorder) [26]. As a matter of fact, repeated ECG in STROKESTOP substantially increased AF detection, since intermittent periodic monitoring allowed a four-fold increased detection of AF as compared with the initial single ECG [26].

Our experience took place outside the traditional settings of medical care and no specific criteria for subjects’ selection were applied, except for the exclusion of patients with an history of AF or a previous pacemaker implant. Our results indicate that the setting of meetings or social recreational activities may be of great interest for AF screening, since in our registry AF was found in a higher proportion of subjects as compared with the screening activities performed during the Belgian Heart week initiative, which found AF in 1.1% of subjects aged at least 20 years with no previous diagnosis of AF [27]. Other studies, using the MyDiagnostick as a screening tool for AF in different settings, found a detection rate of 1.1% in the population aged ≥ 60 years undergoing influenza vaccination [28] and up to 5.5% in patients aged ≥ 65 years admitted to a visit by general practitioners [29]. The variable results reported in literature in terms of frequency of new AF detected, may be mainly related to different study settings and age of targeted population [13,15]. In the literature the age of screened populations showed differences among the various reports, since median age was 58 in the Belgian heart week experience [27], 75 in the randomized trial performed in the Netherlands [30] and 68 in our study. The setting of screening and the age cut-offs appear to be important in conditioning the results of any screening initiative. In a meta-analysis based on 19 AF screening studies based on a single-time point rhythm recording, the rates of AF detection ranged from 0.35% in those enrolling subjects aged ≥ 40 years to 2.34% in studies recruiting older subjects aged ≥ 65 years [13]. Among 138,000 subjects included in the meta-analysis, the pooled yield of screening resulted in a rate of AF detection of 1.44% at age ≥ 65 years, i.e., lower than what we found in our sample [13]. In a recent cluster randomized trial performed in the primary care setting, the active screening arm resulted in detection of AF in 1.62% of subjects aged ≥ 65, with no significant differences versus the AF rate of 1.53% found among subjects in the usual care arm [30]. The lack of benefit found in this study in active screening arm can be explained by the high rate of AF detection that characterizes usual care in The Netherlands, as a result of implemented guidelines for cardiovascular disease management, resulting in a higher baseline prevalence of AF as compared with the SAFE study [31]. To reduce the complexity of screening initiatives, a series of studies evaluated the possibility of an improved patient targeting, by the selection of candidates with a higher likelihood to have AF detected [32]. The CHA_2_DS_2_VASc score, even if not developed for this purpose, has been tested in the form of “virtual” CHA_2_DS_2_VASc, both for screen-detected and incident AF [32]. Our study indicates that a CHA_2_DS_2_VASc > 0 in males and >1 in females, or its analogue CHA_2_DS_2_VA > 0, can be useful risk stratifiers for detecting unknown AF, since associated with very high sensitivity for AF detection in the whole population. Our study found that female sex is negatively associated with AF detection at screening, a finding not stressed in previous meta-analyses [13,15], but considered in some scores used for predicting incident AF in the community [32]. The direct implications of this observation is the opportunity to modify CHA_2_DS_2_VASc, for the purpose of AF screening, into a sex neutral score (CHA_2_DS_2_VASc > 0 in males and >1 in females) or a sex-independent score CHA_2_DS_2_VA > 0). Since a series of clinical factors and comorbidities are included within the CHA_2_DS_2_VASc score this score may have implications for expressing the extent of atrial remodeling that may predispose to AF onset and therefore it is reasonable to explain why higher CHA_2_DS_2_VASc scores are associated with higher rates of AF detection at screening. According to literature, CHA_2_DS_2_VASc scores is directly associated with the incidence of new-onset AF, and has a relatively high performance for AF prediction [33]. Therefore, despite the CHA_2_DS_2_VASc score was proposed for stroke risk stratification in AF, several previous studies found that CHA_2_DS_2_VASc score alone or used in combination with other macroscopic marker of atrial cardiomyopathy (such as left atrial diameter) may improves, at least statistically, the prediction of AF onset or progression [33,34]. In our study no patient younger than 50 was diagnosed with AF and this further underscores the age-dependency of the arrhythmia [13]. Nonetheless, a meta-analysis, including also multiple-time point screening, found that active screening is effective from 40 years of age [15]. We suggest that a CHA_2_DS_2_VASc > 0 in males and >1 in females can be an advisable risk stratifier when considering patients under 65 years, in order to enhance the likelihood of identification of AF patients who can be candidates for oral anticoagulants. It is noteworthy that in the meta-analysis by Lowres et al. [13] 46–54% of the cases of new AF identified by screening at age < 65 had at least 1 non-age or sex-related stroke risk factor, thus supporting the usefulness of some selection criteria based not only on age. The clinical factors that we identified allow to improve patient targeting in screening initiatives, and this may facilitate the organizational aspects, which have to consider as key elements the specificity of the setting, the need for adequate information to potential candidates on the scope and implications of screening, as well as the definition of pathways for direct referral after AF diagnosis to physicians (general practitioners or cardiologists) in charge of clinical evaluation and decision making [22,35]. Our standard for AF diagnosis was the 12-lead ECG, in line with current European guidelines [14] and previous experiences [30,36]. For practical reasons we did not adopt the alternative possibility of AF diagnosis based on a single-lead ECG tracing showing AF with at least 30 s duration, analyzed by an expert in ECG reading [14]. Moreover, a 12-lead ECG is required as the basis for a complete clinical evaluation [37]. More recently, a large number of devices, including wearables have been proposed for AF screening, but their appropriate use still needs to be clarified, also with the need for organizing referral for clinical evaluation in case of suspected or detected AF [16,35,38]. As a consequence of the disruptive effects of COVID-19 pandemic, screening for AF may become even more important in the next future. Indeed, COVID-19 caused a polarization of care on the various manifestation of SARS-CoV-2 infection and on its management [39,40,41] that coupled with the fear and psychological distress diffused within the population [42,43,44,45] led to a marked reduction in access to Emergency Departments for acute cardiovascular conditions, including new-onset AF [46,47,48,49,50]. Some data from large datasets indicate that the risk of undiagnosed AF during COVID-19 lockdown is associated with an increased occurrence of stroke, related to lack of anticoagulation in patients at risk [48], thus making of great clinical value any initiatives of opportunistic screening for detecting unknown and previously undetected AF.

### Limitations

Our study has some limitations since the clinical evaluation and prescription of oral anticoagulants in high risk patients and their follow-up was not part of the study, similarly to other reports in literature focused on detection of unknown AF in specific populations object of screening [27,28,29]. Anyway, all the subjects in our study with a diagnosis of AF as a result of screening were managed according to usual practice, with AF detection followed by direct referral to a cardiologist for a complete clinical evaluation and prescription of anticoagulants when appropriate. According to our methods, we cannot exclude that the delay up to 24 h in performing a 12-lead ECG in case of a red alarm, related to an irregular tracing suspected for AF but not immediately inspected by a cardiologist, could have missed some cases of paroxysmal AF. For practical reasons analysis of ECG tracings derived from MyDiagnostick was not included in the study plan and the device was simply used for its ability to raise the suspicion of AF through its red alarm. Similar to other AF screening initiatives, selection of candidates was based on an interview excluding patients who reported a known history of AF and did not include a direct access to health care records to exclude a previous AF diagnosis.

Our study was not planned for testing the diagnostic performance of MyDiagnostick, which was already defined and reported in the literature with appropriate protocols specifically designed for this purpose [20]. For this reason, we did not perform a 12-lead ECG in case of a green light. We are conscious that we might have missed some false negative cases, but the aim of our study was to assess the efficacy and feasibility of a single-time and opportunistic screening for AF in subjects with no history of AF. The single-time check, the intrinsic characteristics of the device in terms of performance, as well as the delay between a positive screening and the following 12-lead ECG, may imply some missed cases. In any case, we would like to highlight that our project was “population-oriented” and not “device-oriented” so the performance of the MyDiagnostick device was not a specific object of investigation. Our screening was performed in the particular setting of meetings or social recreational activities organized by patient groups or volunteers. This implied that screening was often performed on meeting on Sunday morning or afternoon in rural centers, without hospital facilities close to site of screening. Our aim was to detect undiagnosed AF with a single time check. In order to reduce the potential anxiety of subjects in whom a “red light” was delivered by the MyDiagnostick (as a results of rhythm irregularities possibly related to AF, artifacts, frequent atrial premature beats, etc.), we decided to plan the 12-lead ECG in the following working day and to take direct responsibility of the planning and execution of the 12-lead ECG in our Cardiology Clinic. As repeatedly stressed by the Guidelines [14] and by the US Preventive Task Force [51], anxiety is a major potential drawback and an unpleasant consequence of population screening for AF. Our methods were targeted to avoid referral of the subject with red light at MyDiagnostick to other physicians or to a hospital without taking direct responsibility of 12-lead ECG execution, which was not anyway possible in the setting where screening was performed. According to our Methods the 12-lead ECG was regularly performed within 12–24 h from screening when needed (i.e., in case of red light of My Diagnostick). Our project was “population-oriented” and not “device-oriented” so the performance of the MyDiagnostick device was not tested, also because the delay between testing with MyDiagnostick and 12-lead ECG (up to 24 h) represents a major limitation for an accurate estimate of device performance in terms of sensitivity. Finally, we did not consider the possibility of employing biomarkers for improving patient targeting [52,53], since this approach was not practicable in the setting that we considered.

## 5. Conclusions

The present study highlights that screening for AF, as performed during initiatives promoted by volunteers, patient groups and associations for sensitization on healthy behaviors and wellness, using a single-time point method with a hand-held ECG device of 1 min recording, followed by confirmation on 12-lead ECG, results in detection of AF in 2.0% of the whole screened population and in 2.9% among subjects aged ≥ 65 years. Patient targeting can be improved using as cut-offs for candidates’ selection age or specific values of the CHA_2_DS_2_VASc/CHA_2_DS_2_VA scores. The age threshold of ≥65 years may allow to achieve a high sensitivity (96.4%), but the use of CHA_2_DS_2_VASc > 0 in males and >1 in females, or CHA_2_DS_2_VA > 0 may further increase sensitivity up to 98.2%. Limiting the analysis to subjects aged ≥ 65 years, as currently suggested by many consensus guidelines, a CHA_2_DS_2_VASc > 1 in males and >2 in females, or CHA_2_DS_2_VA > 1, are associated with the highest sensitivity and negative predictive value for AF detection, while age ≥ 75 is associated with a marked drop in sensitivity. The present study also indicates that the setting of meetings or social recreational activities organized by groups of volunteers and associations for promoting healthy behaviors and wellness may be of interest for the scopes of AF screening.

## Figures and Tables

**Table 1:** Baseline characteristics of the whole population, and subjects with a negative and a positive screening for atrial fibrillation (AF), respectively. Data are shown as number (%). The results of univariate comparisons are also shown.

	Total	Screening NEGATIVE for AF	Screening POSITIVE for AF ^1^	OR (95% CI)	*p*
Number of subjects	2814	2758 (98.0)	56 (2.0)		
Female gender	1617 (55.5)	1597 (57.9)	20 (35.7)	0.40 (0.23–0.7)	0.001
Age, years, mean (SD)	66 ± 13	68 ± 9	74 ± 7	1.09 (1.06–1.13)	<0.001
Age, years, median (IQR)	68 (61–74)	69 (61–74)	70-(65–75)		
Age ≥ 65 yrs	1848 (65.7)	1794 (65)	54 (96.4)	14.51 (3.53–59.63)	<0.001
Age ≥ 75 yrs	691 (24.6)	663 (24)	28 (50)	3.16 (1.86–5.37)	<0.001
Age strata (yrs)					
<30	67 (2.4)	67 (2.4)	0		
30–39	60 (2.1)	60 (2.2)	0		
40–49	138 (4.9)	138 (5)	0		
50–59	353 (12.5)	352 (12.8)	1 (1.8)		
60–69	932 (33.1)	921 (33.4)	11 (19.6)		
70–79	988 (35.1)	956 (34.7)	32 (57.1)		
≥80	276 (9.8)	264 (9.6)	12 (21.4)		
Heart failure	100 (3.6)	85 (3.1)	15 (26.8)	11.5 (6.13–21.59)	<0.001
Hypertension	1389 (49.4)	1347 (48.8)	42 (75)	3.14 (1.71–5.78)	<0.001
Diabetes	315 (11.2)	304 (11)	11 (19.6)	1.97 (1.01–3.86)	0.043
Previous AMI	180 (6.4)	169 (6.1)	11 (19.6)	3.75 (1.90–7.37)	<0.001
Peripheral artery disease	218 (7.7)	212 (7.7)	6 (10.7)	1.44 (0.61–3.40)	0.401
Previous stroke/TIA	69 (2.5)	64 (2.3)	5 (8.9)	4.13 (1.59–10.69)	0.002
CHA_2_DS_2_VAsc score mean (SD)	2.3 ± 1.4	2.5 ± 1.3	3.3 ± 1	1.71 (1.45–2.02)	<0.001
CHA_2_DS_2_VAsc score median (IQR)	2 (1–3)	2 (1–3)	3 (3–4)		
CHA_2_DS_2_VAsc score > 0 in males and >1 in females	2163 (76.9)	2108 (76.4)	55 (98.2)	16.96 (2.34–122.79)	<0.001
CHA_2_DS_2_VASc score strata					
0	255 (9.1)	254 (9.2)	1 (1.8)		
1	607 (21.6)	606 (22)	1 (1.8)		
2	767 (27.3)	758 (27.5)	9 (16.1)		
3	655 (23.3)	634 (23)	21 (37.5)		
4	358 (12.7)	345 (12.5)	13 (23.2)		
5	119 (4.2)	112 (4.1)	7 (12.5)		
6	34 (1.2)	32 (1.2)	2 (3.6)		
≥ 7	19 (0.6)	17 (0.6)	2 (3.5)		

^1^ AF confirmed by 12-lead ECG after a suspected recording by MyDiagnostick single-lead ECG. AF: Atrial fibrillation; AMI: Acute myocardial infarction; CI: Confidence interval; SD: Standard deviation, IQR: Interquartile range, OR: Odds ratio; TIA: Transient ischemic attack; yrs: Years.

**Table 2:** Sensitivity, specificity, positive predictive value and negative predictive value of different risk stratifiers’ cut-off values with regard to identification of subjects with screen-detected AF, as confirmed by 12-lead ECG.

	Sensitivity	Specificity	PPV	NPV
	**%**	**%**	**%**	**%**
Age ≥ 65 (1848 [65.7%])	96.4	24.2	2.9	99.8
Age ≥ 75 (691 [24.6%])	50	76	4.1	98.7
CHA_2_DS_2_VASc > 0 in males and >1 in females or CHA_2_DS_2_VA > 0 (2163 [76.9%])	98.2	23.6	2.5	99.8
CHA_2_DS_2_VASc > 1 in males and >2 in females or CHA_2_DS_2_VA > 1 (1510 [53.7%])	92.9	47.1	3.4	99.7
CHA_2_DS_2_VASc > 2 (1185 [42.1%])	80.4	58.7	3.8	99.3
CHA_2_DS_2_VASc > 3 (530 [18.8%])	42.9	81.7	4.5	98.6
CHA_2_DS_2_VA > 2 (781 [27.8%])	73.2	73.2	5.2	99.3
CHADS_2_ > 0 (1708 [60.7%])	92.9	40	3	99.6
CHADS_2_ > 1 (722 [25.7%])	69.6	75.2	5.4	99.2
CHADS_2_ > 2 (196 [7%])	23.2	93.4	6.6	98.4
CHADS_2_65 > 0 (2150 [76.4%])	98.2	24	2.6	99.8
CHADS_2_65 > 1 (1255 [44.6%])	89.3	56.3	4	99.6
CHADS_2_65 > 2 (336 [11.9%])	37.5	88.6	6.3	98.6

Legend: PPV: Positive predictive value; NPV: Negative predictive value.

**Table 3:** For subjects aged ≥ 65 (*n* = 1848) sensitivity, specificity, positive predictive value and negative predictive value of different cut-offs of risk stratifiers, and age ≥ 75, with regard to identification of subjects with screen-detected AF, as confirmed by 12-lead ECG.

	Sensitivity	Specificity	PPV	NPV
	**%**	**%**	**%**	**%**
Age ≥ 75 (691 [37.4%])	51.9	63.1	4.1	97.8
CHA_2_DS_2_VASc > 1 in males and >2 in females or CHA_2_DS_2_VA > 1 (1432 [77.5%])	94.4	23	3.6	99.3
CHA_2_DS_2_VASc > 2 (1141 [61.7%])	81.5	38.9	3.9	98.6
CHA_2_DS_2_VASc > 3 (522 [28.2%])	44.4	72.2	4.6	97.7
CHA_2_DS_2_VA > 2 (767 [41.5%])	75.9	59.5	5.3	98.8
CHADS_2_ > 0 (1406 [76.1%])	94.4	24.5	3.6	99.3
CHADS_2_ > 1 (678 [36.7%])	70.4	64.3	5.6	98.6
CHADS_2_ > 2 (187 [10.1%])	24.1	90.3	7	97.5
CHADS_2_65 > 1 (1211 [65.5%])	90.7	35.2	4	99.2
CHADS_2_65 > 2 (327 [17.7%])	38.9	82.9	6.4	97.8

Legend: PPV: Positive predictive value; NPV: Negative predictive value.

## Data Availability

The data presented in this study are available upon reasonable request from the corresponding author. The data are not publicly available due to privacy restrictions.

## Supplementary Materials

The following are available online at https://www.mdpi.com/2077-0383/10/4/729/s1, Figure S1: ROC curve of age for detecting AF, Table S1: Baseline characteristic of the population aged ≥ 65 years, and of patients with a negative and a positive screening for AF, respectively.
