# Supplementary material for: Clinical Factors Associated with Atrial Fibrillation Detection on Single-Time Point Screening Using a Hand-Held Single-Lead ECG Device
Source: J Clin Med. 2021 Feb 12;10(4):729. doi: 10.3390/jcm10040729 (PMC7917757; doi:10.3390/jcm10040729)
Supplement: Supplementary file 1 [file jcm-10-00729-s001.pdf]

Article

Clinical Factors Associated with Atrial Fibrillation Detection on Single-Time Point Screening Using a Hand-Held Single-Lead ECG Device

Giuseppe Boriani [1,*], Pietro Palmisano [2], Vincenzo Livio Malavasi [1], Elisa Fantecchi [1], Marco Vitolo [1,3], Niccolo' Bonini [1], Jacopo F. Imberti [1], Anna Chiara Valenti [1], Renate B. Schnabel [4] and Ben Freedman [5]

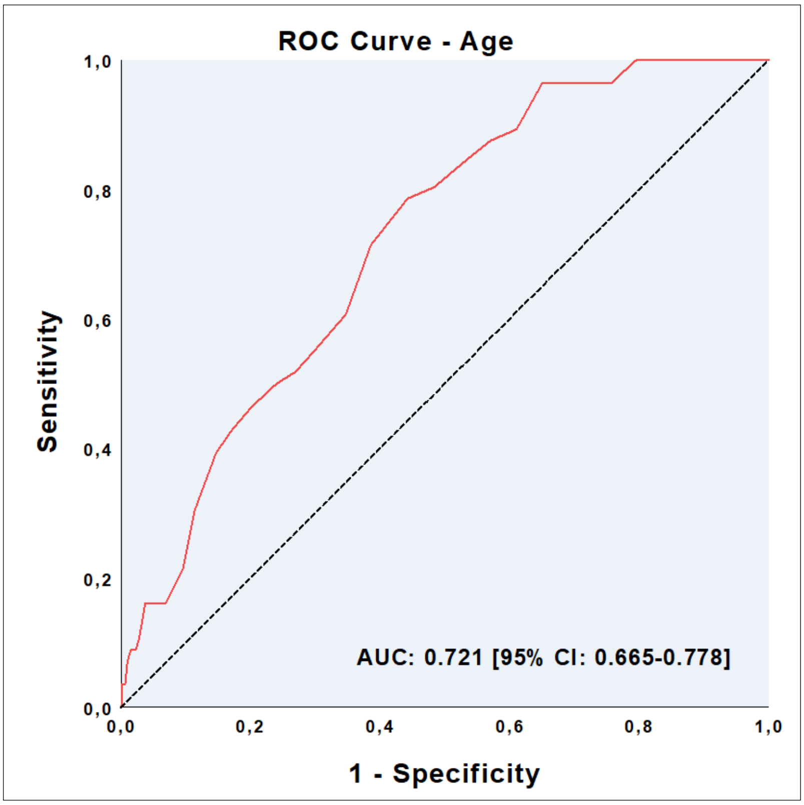

Figure 1. ROC curve of age for detecting AF.

AUC, area under the curve; CI, confidence interval

Table 1. Baseline characteristic of the population aged ≥ 65 years, and of patients with a negative and a positive screening for AF, respectively.

	Total	Screening NEGATIVE for AF	Screening POSITIVE for AF[1]	OR (95% CI)	*p*
Number of subjects	1848	1794	54		
Female gender	1028 (55.6)	1008 (56.2)	19 (35.2)	0.42 (0.24-0.75)	0.002
Age,years, mean (SD)	73±6	73±6	75±7	1.07 (1.01-1.12)	0.003
Age, years, median (IQR)	72 (68-77)	72 (68-77)	75 (70-79)		
Age ≥ 75 yrs	691 (37.4)	663 (37)	28 (51.9)	1.84 (1.07-3.16)	0.026
Age strata					
65-69	584 (31.6)	574 (32)	10 (18.5)		
70-79	988 (53.5)	956 (53.3)	32 59.3)		
≥ 80	276 (14.9)	264 (14.7)	12 (22.2)		
Heart failure	82 (4.4)	68 (3.8)	14 (25.9)	8.88 (4.61-17.1)	< 0.001
Hypertension	1119 (60.6)	1077 (60)	42 (77.8)	2.33 (1.22-4.46	0.009
Diabetes	259 (14)	248 (13.8)	11 (20.4)	1.59 (0.81-3.13)	0.172
Previous AMI	149 (8.1)	139 (7.7)	10 (18.5)	2.71 (1.33-5.49)	0.004
Peripheral artery disease	191 (10.3)	185 (10.3	6 (11.1)	1.09 (0.46-2.58)	0.849
Previous stroke/TIA	62 (3.4)	57 (3.2)	5 (9.3)	3.11 (1.19-8.1)	0.014
CHA_2DS_2VASc score mean (SD)	3±1.2	2.9±1.2	3.2±1.3	1.32 (1.01-1.71)	0.040
CHA_2DS_2VASc score median (IQR)	3 (2-4)	3 (2-4)	3 (3-4)		
CHA_2DS_2VASc score strata					
1	133 (7.2)	132 (7.4)	1 (1.9)		
2	574 (31)	565 (31.5)	9 (16.7)		
3	619 (33.5)	599 (33.4)	20 (37)		
4	351 (19)	338 (18.8)	13 (24.1)		
5	119 (6.4)	112 (6.2)	7 (13)		
6	34 (1.8)	31 (1.7)	3 (4.1)		
≥ 7	20 (1.1)	17 (0.9)	3 (4.1)		

[1]AF confirmed by 12-lead ECG after a suspected recording by MyDiagnostick single-lead ECG. AF: atrial fibrillation; AMI: acute myocardial infarction; CI: confidence interval; SD: standard deviation, IQR: interquartile range, OR: odds ratio; TIA: transient ischaemic attack; yrs: years .
